# Targeting T Cell Metabolism in Inflammatory Skin Disease

**DOI:** 10.3389/fimmu.2019.02285

**Published:** 2019-09-24

**Authors:** Leonhard von Meyenn, Nicole Leonie Bertschi, Christoph Schlapbach

**Affiliations:** Department of Dermatology, Inselspital, Bern University Hospital, University of Bern, Bern, Switzerland

**Keywords:** T cell metabolism, inflammatory skin disease, glycolysis, OXPHOS, lipid metabolism

## Abstract

A properly functioning T cell compartment is crucial to protect the host from infections, tumors, and environmental substances. In recent years, it has become increasingly clear that the processes underlying proper T cell activation, proliferation, and differentiation require well-tuned and dynamic changes in T cell metabolism. Thus, proper metabolic reprogramming in T cells is crucial to ensure proper immunity in the context of infection and anti-tumor immunity. Conversely, aberrant regulation of T cell metabolism can impair T cell function and thereby contribute to T cell-mediated disease. In this review, the relevance of recent insights into T cell metabolism for prototypical T cell-mediated skin diseases will be discussed and their therapeutic potential will be outlined. First, the major modules of T cell metabolism are summarized. Then, the importance of T cell metabolism for T cell-mediated skin diseases such as psoriasis and allergic contact dermatitis is discussed, based on the current state of our understanding thereof. Finally, novel therapeutic opportunities for inflammatory skin disease that might emerge from investigations in T cell metabolism are outlined.

## Introduction

T cells are key players of the adaptive immune system by acting as potent effectors and regulators of immunity. Adaptive T cell immunity results from clonal expansion of antigen-specific T cells that were activated by their cognate antigen presented by dendritic cells in secondary lymphoid organs (1). After priming of naive T cells, multiple subsets of effector and memory T cells with different phenotypic and functional properties are generated. Effector T cells (T_EFF_) migrate to peripheral tissues and inflamed sites to fight infection. After successful clearance of the invading antigen, the vast majority of effector cells die, leaving behind a small population of long-lived memory T cells (2). In case of re-exposure to previously encountered pathogens or antigens, memory T cells provide rapid and effective protective immunity.

Based on their migratory properties, memory T cells can be categorized in three major subsets: Central memory T (T_CM_) cells, effector memory T (T_EM_) cells, and the recently identified tissue-resident (T_RM_) cells. T_CM_ cells express the chemokine receptor CCR7 and the vascular addressin L selectin (CD62L), enabling them to circulate between lymph nodes and blood. T_EM_ cells express tissue-homing receptors that grant them access to peripheral tissues. Skin-homing T_EM_, for instance, express the E-selectin ligand Cutaneous Lymphocyte Antigen (CLA), which allows them to enter the skin, whereas the integrin α4β7 permits gut-homing T_EM_ to enter the gut (3). T_RM_ cells are non-recirculating memory T cells that persist long-term in epithelial barrier tissues and provide rapid front-line immune protection in peripheral tissues (4).

It is now increasingly understood that T cell identity and function are intertwined with metabolic pathways that are required for energy and substrate generation on the one hand, but also act to regulate effector functions, differentiation, and gene expression on the other hand (5). In this review, we will first summarize the current literature on T cell immunometabolism. Then, we will explore how the cutaneous environment in inflammatory skin disease might shape and influence T cell function via the modulation of T cell metabolism and look ahead on therapeutic opportunities that might arise therefrom.

## Modules of T Cell Metabolism

### Glycolysis

**Figure 1:**
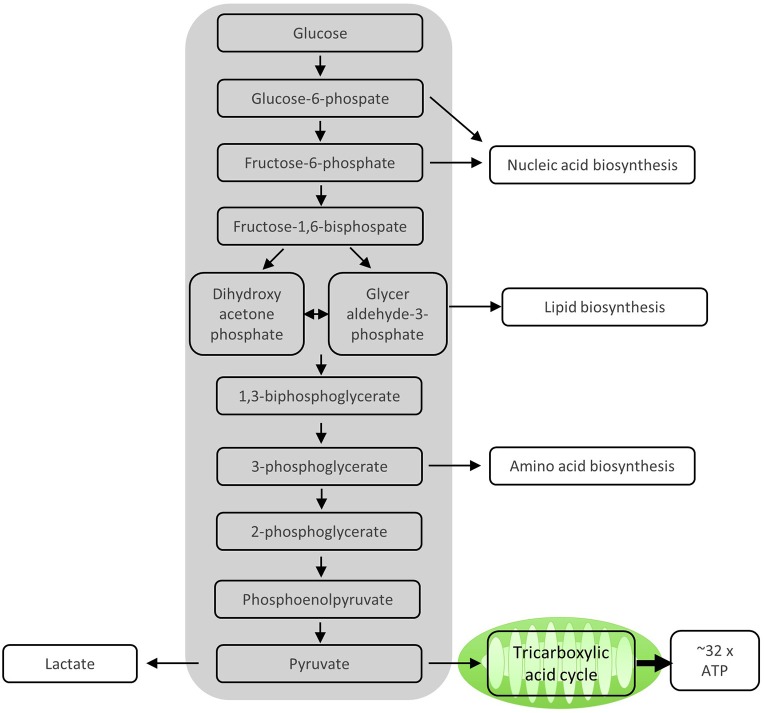
Enzymatic steps of glycolysis. During the 10 enzymatic steps of glycolysis in which glucose is converted to pyruvate, important intermediate molecules for nucleic acid synthesis, lipid biosynthesis, and amino acid biosynthesis, all of which are important for proliferating T cells, are generated. The sequential enzymatic reactions of glycolysis occurring in the cytosol generate ATP. The remaining pyruvate can be oxidized in the mitochondria via the tricarboxylic acid cycle and subsequent oxidative phosphorylation (OXPHOS) to generate ATP. Alternatively, pyruvate can be reduced to lactate and excreted, which mainly happens in proliferating T cells.

**Figure 2:**
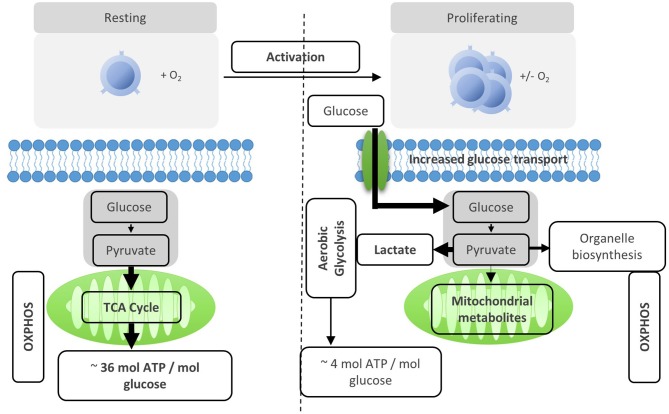
Activation reprograms T cells to increased usage of aerobic glycolysis. Resting T cells engage OXPHOS and glycolysis to generate energy for homeostasis and survival. Upon activation, T cells are reprogramed to increase aerobic glycolysis. The relative increase of anaerobic glycolysis after activation provides less energy, but generates molecules for biosynthesis.

After activation by cognate antigen, T cells rapidly increase their glucose uptake by upregulating the cell surface expression of glucose transporters such as GLUT1 (6). Once in the cytosol, glucose immediately enters the glycolysis pathway in which glucose is metabolized to pyruvate by a series of enzymatic reactions (Figure 1). At the same time, lactate excretion increases as a consequence of enhanced conversion of pyruvate to lactate, indicating that T cells engage in aerobic glycolysis (Figures 1, 2). Aerobic glycolysis describes the reduction of pyruvate to lactate in the presence of oxygen, instead of its oxidative phosphorylation (OXPHOS) in the mitochondria via the tricarboxylic acid (TCA) cycle. This process of aerobic glycolysis is also referred to as the “Warburg effect” in cancer cells (7). While aerobic glycolysis generates less ATP per molecule of glucose than OXPHOS, aerobic glycolysis yields metabolic intermediates important for cell growth and proliferation, and contributes to the cellular redox balance (NAD^+^/NADH) (8). During the 10 enzymatic steps of glycolysis in which glucose is converted to pyruvate, a variety of intermediates are generated that are used in different biosynthetic pathways. These include the pentose phosphate pathway, serine biosynthesis, *de novo* fatty acid synthesis, and hexosamine biosynthesis. The pentose phosphate pathway, for instance, donates important precursors for nucleotide synthesis and is thus of crucial importance for cell growth (9–11). As a consequence, glycolysis is not only an energy-generating pathway for T cells, but also a metabolic basis for anabolic biosynthesis and proliferation (10).

The rapid changes in T cell metabolism after activation are regulated by a number of transcription factors and signaling pathways (8). Signals from cytokines such as IL-2 lead to upregulation of nutrient transporters. Ligation of the co-stimulatory molecule CD28 induces phosphatidylinositol-3-kinase (PI3K)-dependent phosphorylation of Akt (12, 13). Phosphorylated Akt induces expression and translocation of glucose transporters to the cell surface, enhances activity of glycolytic enzymes and activates key metabolic regulators such as mTOR (14). mTOR is a central orchestrator of T cell metabolism and is crucial for upregulation of glycolysis in activated T cells (15) (Figure 2). Further transcription factors involved in regulating activation-induced glycolysis are c-Myc, estrogen receptor α (ERRα) and hypoxia inducible factor-1α (HIF-1α) which coordinately drive the expression of genes involved in metabolism to fuel the bioenergetic needs of clonal expansion (8, 16–18).

Glycolytic activity in T cells is crucial for effector functions in T cells. This is best described for interferon-γ (INF-γ) production of CD8^+^ and CD4^+^ T_EM_ cells, with multiple mechanisms linking glycolysis to INF-γ production. For instance, activation-induced glycolysis is accompanied by increased activity of GAPDH which promotes chromatin remodeling at the IFNG locus (15). In resting T cells, on the other hand, GAPDH is not engaged in glycolysis but is able to inhibit translation of IFNG mRNA by binding to its untranslated region (19). More broadly, glycolysis has been linked to pathogenic effector T cell responses in a variety of disease models, such as allogeneic transplantation (20), experimental autoimmune encephalitis (21), and systemic lupus erythematosus (SLE) (22). In these models, inhibiting glycolysis in T cells ameliorated the disease (21), suggesting that manipulation of glycolysis represents a promising target for T cell mediated disease. However, the role of T cell metabolism seems to be context and disease dependent, because reduced glycolysis has also been reported to play a pathogenic role in other disease settings, such as rheumatoid arthritis (23). In contrast to effector T cells, the importance of glycolysis for regulatory T cells (T_REG_) is less well-understood. Depending on the setting within which T_REG_ cells have been investigated, glycolysis appears to be necessary or not for their suppressive function (24–26). Taken together, well-coordinated glycolytic activity is crucial for proper effector functions in T cells and represents an intriguing leverage point for therapeutic intervention even though the precise conditions for this remain to be elucidated.

### Tricarboxylic Acid Cycle

**Figure 3:**
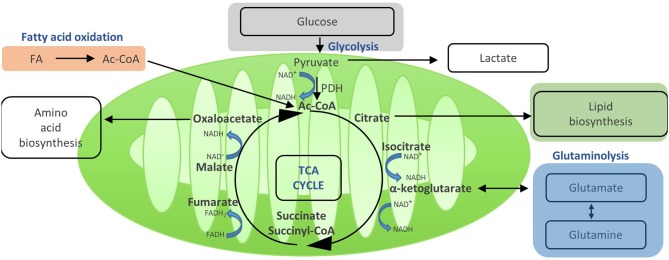
Acetyi-CoA enters the TCA and yields reduced electron carriers (NADH and FADH2) that drive OXPHOS. Once in the mitochondria, pyruvate is converted to acetyl-GoA by pyruvate dehydrogenase, and then enters the TGA cycle. In the TGA cycle, acetyl-GoA is oxidized to carbon dioxide and water, which generates GTP and reduces NAD+ and FADH, the electron carriers that later fuel OXPHOS. In addition, the TGA cycle generates a number of metabolic intermediates for anabolic processes such as amino acid and lipid biosynthesis. Other substrates can also be metabolized in the TGA cycle, such as glutamine via glutaminolysis or fatty acids via oxidation. Thus, the TGA cycle integrates and mediates the metabolism of glucose, amino acids, and fatty acids.

**Figure 4:**
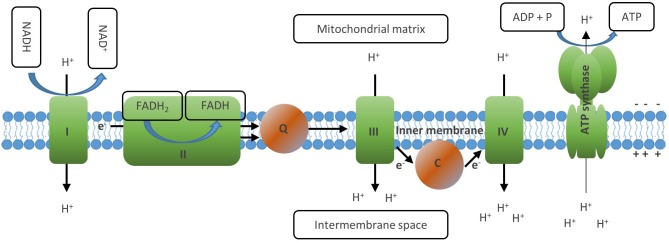
The electron transport chain generates ATP via oxidative phosphorylation. The ETC consists of a series of transmembrane proteins located on the inner mitochondrial membrane (IMM). Complex I and II of the ETC oxidize NADH and FADH2, respectively. Then complex II donates the electrons obtained to complex Ill *via* Coenzyme Q and later to complex IV via cytochrome C, where finally they are transferred to molecular oxygen which generates water. This process is linked to the transfer of protons into the intermembrane space of the mitochondria, which creates an electrochemical proton gradient between the opposite sides of the membrane. The last member of the ETC, the ATP synthase, finally uses this membrane potential to phosphorylate ADP to ATP, thus letting protons back to the mitochondrial matrix. Through this process, OXPHOS mediates maximal usage of the energy stored in nutrient molecules such as glucose or fatty acids by generating 30–36 ATP molecules from one molecule of glucose and 106 ATP from one molecule of palmitate. Full oxidation of NADH and FADH2 generated in the TCA, however, requires the presence of molecular oxygen to serve as acceptor of the electrons generated by the ETC reactions.

Activation of T cells also leads to an increase in their mitochondrial function. Even in the presence of ongoing aerobic glycolysis, enough pyruvate is generated to enter the mitochondria via the mitochondrial pyruvate carrier and fuel the TCA cycle (14) (Figure 3). This process generates further molecules for biosynthesis and drives OXPHOS and, thereby, efficient ATP production. Once in the mitochondria, pyruvate is converted to acetyl-CoA by pyruvate dehydrogenase, and then enters the TCA cycle. In the TCA cycle, acetyl-CoA is oxidized to carbon dioxide and water, which generates GTP and reduces NAD and FADH, the electron carriers that later fuel OXPHOS (14). In addition, the TCA cycle generates a number of metabolic intermediates for anabolic processes such as amino acid and lipid biosynthesis (27). Other substrates can also be metabolized in the TCA cycle, such as glutamine via glutaminolysis or fatty acids via β oxidation (8). Thus, the TCA cycle integrates and mediates the metabolism of glucose, amino acids, and fatty acids (Figure 4).

In terms of T cell function, the TCA cycle appears to be critical for proper T cell activation, proliferation, and production of effector molecules (14). Fatty acid oxidation (FAO) has been shown to be particularly active and important in T_REG_ (24, 28), memory T cells, and in skin-resident T_RM_ (29). Glutaminolysis, the process of breaking down glutamine to α-ketoglutarate which then enters the TCA cycle, has equally been observed to be crucial for T cell function (8). After activation, T cells upregulate their expression of glutamine transporters, increase glutamine uptake, and promote expression of enzymes required for glutaminolysis (13, 16, 30, 31). While glycolysis-derived pyruvate is a critical substrate for oxidative phosphorylation, these “alternative” substrates described above seem to be particularly important to fuel the TCA and thereby T cell function when there is limited availability of glucose (32). The importance of the TCA cycle, FAO, or glutaminolysis for T cell function is exemplified by studies showing that interference with either FAO or glutamine metabolism has the potential to alleviate pathology in T cell-mediated diseases (20, 33, 34).

### Oxidative Phosphorylation

An important function of the TCA cycle is to generate the electron carriers NADH and FADH_2_ by reducing NAD+ and FADH, respectively. These molecules are then oxidized at the inner membrane of the mitochondrion, which drives the electron transport chain (ETC) and finally generates ATP (Figure 4) (14). The ETC consists of a series of transmembrane proteins located on the inner mitochondrial membrane (IMM). Complex I and II of the ETC oxidize NADH and FADH_2_, respectively, and then donate the electrons obtained to complex III and later to complex IV, where they are finally transferred to molecular oxygen which generates water (10). This process is linked to the transfer of protons into the intermembrane space of the mitochondria, which creates an electrical charge between the opposite sides of the membrane. The last member of the ETC, the ATP synthase, finally uses this gradient to phosphorylate ADP to ATP, thus transferring protons back to the mitochondrial matrix. Through this process, OXPHOS mediates maximal usage of the energy stored in nutrient molecules such as glucose or fatty acids by generating 30–36 ATP molecules from one molecule of glucose and 106 ATP from 1 molecule of palmitate (10, 35). Full oxidation of NADH and FADH_2_ generated in the TCA, however, requires the presence of molecular oxygen to serve as acceptor of the electrons generated by the ETC reactions.

A cell's mitochondrial mass determines the amount of OXPHOS a cell can engage in. In response to metabolic stress, cells can increase their mitochondrial mass in a process referred to as mitochondrial biogenesis (36). In line with their increased functional repertoire, memory T cells feature increased mitochondrial mass and function when compared to naive T cells (14, 37, 38). This is represented by a higher spare respiratory capacity (SRC) of memory T cells. SRC reflects a cell's ability to increase OXPHOS for energy generation in response to metabolic stress or increased demand as present under hypoxic conditions, for instance (39). Thus, memory T cells are able to sustain ATP levels and thereby cellular function even under hypoxia. It is thought that this represents a metabolic adaptation to the challenges of immune surveillance in peripheral tissues under inflammatory conditions where nutrients and oxygen are scarce (14). Increased respiratory capacity of memory T cells is at least in part related to increased FAO in the mitochondria (40). T cells with defects in FAO have impaired memory T cell formation, whereas enhanced FAO leads to increases in SRC and memory T-cell generation (37). In circulating memory T cells, the lipids that fuel FAO seem to derive from triacylglycerols that are synthesized in the endoplasmic reticulum from glucose-derived carbon, and not from fatty acid uptake from the environment (41). Conversely, skin T_RM_ seem to be specialized to take up fatty acids from the cutaneous environment and use them for FAO, which is crucial for their function and survival (29). More broadly, memory T cells critically depend on highly functional mitochondria for the mounting of recall responses, for rapid proliferation, calcium signaling, and effector function (14). It is thus not surprising that impaired mitochondrial function leads to decreased T-cell functionality, whereas promotion of mitochondrial function promotes memory T-cell generation and function in a number of infectious models and in T cell mediated diseases (14, 22, 42–44).

### Amino Acid Metabolism

In addition to glucose, amino acids are crucial nutrients for T cells as well (8). Adequate supply of amino acids is critical for T cell growth and function. Amino acids are essential building blocks for protein synthesis, provide an important backbone for *de novo* nucleotide synthesis, and act as metabolic fuel source that can be fed into multiple pathways (45, 46). Therefore, T cells upregulate amino acids transporters and increase their uptake of amino acids immediately after activation (30, 31) which is an important step in the metabolic reprograming of T cells. Influx of branched chain amino acids such as leucine lead to activation of the mTORC1 pathway which is a key event in metabolic programing of T cells.

In agreement with a central role of amino acid metabolism in T cell biology, modulation of amino acid levels either by downregulation of transporters or by limiting amino acid availability strongly affects T cell activation and function (31, 47). For instance, T helper cells deficient for LAT1 (Slc7a5), a transporter for phenylalanine, tyrosine, leucine, arginine and tryptophan, are unable to differentiate into T_H_1 or T_H_17 cells, whereas T reg cell differentiation is still functional (31). This impaired amino acid metabolism has been linked to reduction in TORC1 activity and lower expression of Myc, which in turn impaired the upregulation of the metabolic machinery required for differentiation in activated T cells. Accordingly, therapeutic manipulation of amino acid metabolism such as glutamine metabolism has been shown to inhibit unwanted T cell responses in animal models of allograft rejection (20). Yet, it appears that manipulating amino acid metabolism alone is not sufficient to prevent allograft rejection, since other metabolic pathways had to be inhibited in parallel to achieve adequate T cell inhibition. Interestingly, however, inhibiting amino acid metabolism had reciprocal effects on T regs as compared to effector T cells, as these two T cell subsets differentially depend on it. As a consequence, interference with amino acid metabolism has the potential to generate antigen-specific regulatory T cells while impairing pathological effector T cell responses. Furthermore, amino acids may also directly affect T cell metabolism and function. Elevating L-arginine levels has been shown to induce a shift from glycolysis to oxidative phosphorylation in activated T cells. This was associated with enhanced generation of central memory-like cells with higher survival and function (48).

### Cholesterol and Lipid Metabolism

Lipids and fatty acids make up another group of crucial T cells substrates. They are important building blocks for cell membranes, store high amounts of energy, and provide substrates for cell signaling and post-translational modifications (8). Not surprisingly, T cells therefore induce cellular lipid biosynthesis pathways as an integral part of their activation program (14). These processes are coordinated, at least in part, by c-Myc and mTOR and transcriptionally regulated by sterol regulatory element-binding protein (SREBF) transcription factors (16, 49, 50). SREBFs transcription factors induce gene expression of enzymes and regulators of fatty acid synthesis and of the mevalonate pathways, which are involved in synthesis of cholesterol. Consequently, impairment of SREBF function or other regulators of cholesterol homeostasis leads to failure of T cells to efficiently proliferate (50, 51). Furthermore, the mevalonate pathway may also affect T cell differentiation and function, either by providing cholesterol precursor or indirectly via provision of isoprenoids (52).

Fatty acid synthesis also has important effects on T cell function. Activated T cells can take up fatty acids from the extracellular space or synthesize them *de novo*. Interestingly, different T cell subsets appear to rely differentially on *de novo* synthesized fatty acids. In particular, inhibition of fatty acid synthesis limits T_H_17 cell differentiation while promoting generation of T_REG_ cells and these effects translated into improved disease outcomes in experimental autoimmune encephalomyelitis (EAE) (8, 28). However, even when T cells have defective *de novo* fatty acid synthesis (because of inhibition/deletion of ACC1 for instance), they can compensate for the lack of fatty acid synthesis if there are abundant amounts of fatty acids in the extracellular space, likely through fatty acid uptake (53). Indeed, skin-resident CD8^+^ T_RM_ have a highly effective lipid uptake machinery and are able to metabolize fatty acids from the cutaneous environment via β-oxidation to support their long-term survival and function in the skin (29, 54). In addition, non-T_RM_ effector T cells have been shown to engage in fatty acid oxidation, although the amount of FAO that occurs in effector T cells seems to be highly context dependent (8, 32).

### Regulation of T Cell Metabolism

The metabolic modules described above have to be finely tuned in order to support the diverse metabolic needs of T cells during different states of activation and memory formation. The metabolic pathways preferably used by T cells based on their activation and differentiation status are summarized in Table 1. Failure to adequately meet the demand in energy, biomolecules and other metabolic mediators leads to T cell dysfunction and may contribute to disease (14). Although the regulatory mechanisms that orchestrate these complex metabolic processes are incompletely understood, two metabolic regulators have been found to be central regulators of T cell metabolism: mTOR and AMPK (8).

**Table 1:** Metabolic pathways of T cell subsets according to differentiation and activation status.

**T cell subset**	**Anaerobic glycolysis**	**Aerobic glycolysis**	**Fatty acid oxidation**	**Fatty acid synthesis**	**OXPHOS**
Naive T cell	+	++	++	–	++
Early activated T cell	++	+++	–	+++	+
Late activated T cell	++	+	+++	–	++
Memory T cell	++	+	++	++	++
T_RM_	N.D.	+	+++	N.D.	+++

*Adapted from Veldhoen et al. (55)*.

Mechanistic target of rapamycin (mTOR) has emerged as a central integrator of environmental signals and cellular metabolic state to regulate T cell development, activation, and differentiation. The mTOR protein is a serine/threonine kinase that can form two different complexes, mTORC1 and mTORC2, depending on the specific scaffolding proteins it associates with (10). The different mTOR complexes perform different functions and are regulated by different stimuli. mTORC1 senses the availability of factors that are necessary for cell growth and cell division and links this information with the appropriate metabolic processes. Signals capable of activating mTORC1 are amino acids, oxygen, energy availability, and growth factors (56–59). In situations that are unfavorable for cell growth or division (low ATP levels, hypoxia, etc.), mTORC1 is not active and cell growth is inhibited. mTORC2, on the other hand, is stimulated by growth factors and cytokines while being irresponsive to nutrient-availability (10, 60). mTORC2 primarily regulates metabolism in support of cell survival and the cytoskeleton (60).

AMP-activated protein kinase (AMPK) acts to counterbalance the effects of mTOR. AMPK is an enzyme that functions as a sensor of cellular energy levels. It is activated by high AMP/ATP ratios, which result from shortage of nutrients or other forms of cellular stress. Consequently, activated AMPK downregulates energy consuming pathways such as biomolecule synthesis while upregulating those that generate energy, such as oxidation of glucose and fatty acids (10, 61). Mechanistically, AMPK has been shown to inhibit activation of mTOR (62). Given its central role in inhibiting cellular anabolism, it is not surprising that activation of AMPK suppresses the differentiation of T cells, as shown in a number of studies (10, 63–65). It has been proposed that activation of AMPK might specifically impair differentiation of T_H_1 and T_H_17 cells and that this effect is beneficial in animal models of T cell mediated inflammatory disease (10). However, in most of these study, it has not been addressed to what extend these AMPK-mediated effects are primarily T cell intrinsic or whether they include T cell independent effects of AMPK modulation. Furthermore, inhibitory effects of AMPK on T cell differentiation appear to depend on the metabolic environment. In fact, AMPK has been shown to be dispensable for T cell differentiation under normal metabolic conditions, since T cells which lack a functional AMPK are still able to acquire effector functions but fail to differentiate under suboptimal environmental conditions (32, 66). Taken together, AMPK might thus primarily function to mediate T cell adaptation to situations of low nutrient availability as encountered in hypoxic and glucose-deprived microenvironments of peripheral tissues under inflammatory conditions.

### T Cell Metabolism–Conclusions

In recent years, research on T cell metabolism has attracted great attention and generated substantial insights into the intricate links between T cell function and T cell metabolism. Although many aspects of the complex mechanisms that link these central aspects of T cell biology remain to be elucidated, it is unquestioned that the therapeutic manipulation of T cell metabolism holds great potential in immune mediated disease. It seems possible that 1 day, overactive T cells in inflammatory disease can be curbed and exhausted T cells in cancer can be activated through manipulation of their metabolism. Before such therapies can be developed, however, many obstacles remain to be overcome, one of which is the fact that metabolism is a central function of all cells. Developing means to specifically target T cells will thus be a key step on the way to metabolism based T cell manipulation in health and disease.

## T Cell Metabolism in Inflammatory Skin Disease

### Effects of the Metabolic Microenvironment on T Cell Function

As outlined above, a T cell's ability to survive, proliferate, and exert effector functions is highly dependent on its metabolic state. In turn, a T cell's metabolic state is highly dependent on the microenvironment, since it is the source from which T cells take up the nutrients they require for their proper functioning (32, 67). Studies in the field of cancer immunology have established that limited nutrient availability in the microenvironment can profoundly affect T cell effector functions (68). Conversely, high concentrations of tumor derived metabolites such as lactate can hinder activation of tumor-infiltrating T cells (69, 70). Although there is a lack of studies actually quantifying the metabolic microenvironment in inflammatory skin disease, it is likely that these interactions between T cells and the cutaneous environment of inflamed skin are equally important for T cell function.

Secondary lymphoid tissues such as the lymph nodes provide a nutrient-rich environment optimal for survival, activation, and growth of T cells (71, 72). However, once T cells migrate into peripheral tissues like the skin, they are forced to metabolically adapt to the local conditions, which may differ substantially in terms of nutrient availability (55). It is assumed that the microenvironment of the skin is restricted in many nutrients, particularly glucose, but is rich in lipids. While this is a reasonable assumption to make, based on the anatomy and biochemical make-up of the skin, it is important to note that cutaneous interstitial glucose, amino acid, or fatty acid levels have never been quantified comprehensively, neither under homeostatic nor under inflammatory conditions (73, 74). Future studies are needed to address this in more detail. At any rate, it is likely that T cells encounter a metabolic environment in the skin that is very different from that in blood or in lymphoid tissues and therefore have to adapt metabolically.

An outstanding example of such a metabolic adaptation is the specialization of skin T_RM_ for uptake and β-oxidation of exogenous free fatty acids to support their long-term survival and function (29). The skin is a primary interface between the body and the environment and thus represents a site of first line defense against microbial pathogens, physical damage, and chemical harm (54). This “immunological hotbed” is populated by large numbers of sessile, non-migratory skin-resident T cells (skin T_RM_) which function to provide rapid on-site immune protection against known pathogens (4). Besides the skin, T_RM_ cells are also found in all other major epithelial barrier tissues, such as the lung and the gastro-intestinal tract (55, 75, 76). In a landmark study by Pan et al., the transcriptional program of CD8^+^ T_RM_ in skin was determined before and after their generation by vaccination with vaccinia virus (skin scarification). Strikingly, CD8^+^ T_RM_ upregulated a transcriptional program that was strongly enriched for genes associated with uptake and metabolism of exogenous free fatty acids as they differentiated into bona fide T_RM_ 90 days post-vaccination. Amongst the top upregulated genes were fatty acid binding proteins 4 and 5 (Fabp4/5), Cd36, and lipoprotein lipase (Lpl), all of which are genes involved in lipid uptake and fatty acid metabolism. The lipoprotein lipase Lpl cleaves triglycerides into free fatty acids and diacylglycerol. Cd36 is a lipid scavenger receptor able to bind and internalize free fatty acids. Fatty acid binding proteins are intracellular chaperones able to shuttle FFAs from the cytoplasm to the mitochondria for β-oxidation. This transcriptional signature of T_RM_ was further confirmed to have important functional relevance. T_RM_ were shown to have a higher capacity to take up free fatty acids from the environment and a higher capacity to perform mitochondrial fatty acid β-oxidation when compared to T_CM_ or T_EM_. This was crucial for the proper functioning of T_RM_, as inhibition of these metabolic pathways resulted in decreased generation, persistence and function of T_RM_ in the skin. For instance, pre-treatment of CD8^+^ T cells with etomoxir, an irreversible inhibitor of carnitine palmitoyltransferase 1 (CPT1a) which catalyzes the rate limiting step for mitochondrial fatty acid β-oxidation, strongly impaired the generation and maintenance of skin T_RM_.

In the study by Pan et al., the expression of Fabp4/5 in T_RM_ was shown to be regulated by peroxisome proliferator-activated receptor gamma (PPARγ) (29). Pharmacological inhibition or genetic deletion of PPARγ in T cells resulted in decreased generation and function of T_RM_ post-skin scarification. Interestingly, PPARγ is also increasingly implicated in the regulation of “pathogenic” T_H_2 cells in both humans and mice (77). Pathogenic T_H_2 cells are a subset of T_H_2 cells that contain all allergen-specific T_H_ cells in humans (78), provide superior protection in models of parasite infection (79) and mediate tissue pathology in models of allergic inflammation (80). In addition to their dependence on PPARγ, pathogenic T_H_2 cells have been shown to express high levels of IL-9 (78). Intriguingly, IL-9 is a cytokine that is preferentially expressed by skin-homing and skin-resident T_H_ cells (81, 82). Furthermore, this population of IL-9^+^ T_H_ cells was recently found to express high levels of PPARγ which in turn acts as positive regulator of IL-9 expression in these cells (83). These PPARγ^+^ IL-9^+^ T_H_ cells were further found to infiltrate lesions of allergic contact dermatitis, a prototypical T_RM_ mediated disease (84). Taken together, PPARγ emerges as an important regulator of both tissue resident and pathogenic T cells. The underlying reason for this link might be that PPARγ confers a metabolic advantage to T cells that enter the cutaneous microenvironment by mediating metabolic adaptation. Given that PPARγ is a well-established drug target, it appears possible that both CD8^+^ and CD4^+^ T_RM_ will 1 day be targeted via this metabolically relevant transcription factor (85).

Tissue inflammation is intricately linked to hypoxia (86). Hypoxia is a strong inducer of metabolic reprogramming in T cells and activation of the hypoxia response has been shown to cause T cell dysfunction and contribute to disease (87). In particular, hypoxia drives glycolysis to maintain ATP production, thus compensating for reduced OXPHOS activity under conditions of limited oxygen pressure (87). This process is critically regulated by the transcription factor HIF-1α, whose activation induces the expression of glycolytic enzymes such as hexokinase and phosphofructokinase, to ramp up glycolysis (see also section Regulation of T Cell Metabolism) (5, 88). Overactive glycolysis, in turn, has been identified as a hallmark of pathogenic T cells in inflammatory and autoimmune disease (20–22, 67). Activation of HIF-1α may also directly affect the balance between pathogenic T_H_17 cells and regulatory T_REG_ cells. HIF-1α directly binds FOXP3 and promotes its proteasomal degradation, thereby impeding T_REG_ differentiation. Conversely, HIF-1α directly activates RORγt and thus promotes IL-17 expression and T_H_17 differentiation (89). Addressing the hypoxia response in inflammatory skin disease might thus represent a promising new leverage point for therapy. In fact, certain drugs currently used to treat inflammatory skin disease may act via the modulation of the hypoxia response in T cells. For instance, dimethyl fumarate (DMF), used in the treatment of psoriasis, is thought to impair the HIF-1α-induced response by promoting its degradation (90). Inhibition of glycolysis by 2-Desoxy-D-glucose (2-DG) or other inhibitors of glycolysis might further help to restore the balance between T_H_17 and T_REG_ cells in skin diseases such as psoriasis.

### Metabolic Competition Between Different Cell Types in the Skin

Just as T cells themselves, other cells of the skin organ also critically depend on nutrients for their functioning. Innate immune cells (91, 92), keratinocytes (93), endothelial cells (94, 95), stromal cells (96), amongst others, thus all engage in a competition for metabolites with T cells and thereby establish a bidirectional metabolic relationship (55). This competition will be especially active under inflammatory conditions, where cells proliferate and undergo metabolic reprograming. For instance, keratinocytes critically depend on increased glucose uptake via the glucose transporter Glut1 for injury- or inflammation-associated keratinocyte proliferation (93). Consequently, inactivation of Glut1 in keratinocytes decreased psoriasis-like inflammation in mouse models and in human psoriatic skin organoids. Interestingly, when Glut1 was uniquely deleted in keratinocytes by means of conditional knock-out, the immune response in the skin in animal models of psoriasis was not abrogated. Conversely, topical inhibition of glucose transport using a chemical GLUT1 inhibitor inhibited not only keratinocyte proliferation but also prevented infiltration and activation of immune cells. This discrepancy between genetic deletion of GLUT1 in keratinocytes and cell type-independent pharmacological inhibition of Glut1 suggests that this glucose transporter plays a crucial role in the facilitation of the immune response in psoriasis. Thus, inhibiting GLUT1 on T cells (and other immune cells) may be a highly effective therapeutic approach in psoriasis, given that GLUT1 is critical for T cell effector function (6). In fact, there is evidence that regulating glycolysis in T cells might be used to modulate autoimmunity. Overexpression of Glut1 in murine T cells boosts activation-induced production of IL-2 and IFN-γ. In mice carrying such T cells, accumulation of highly activated memory T cells was observed in conjunction with high levels of circulating IgG and deposit of IgG in the kidney. This suggests that Glut1 overexpression in T cells alone may contribute to autoimmunity (12). Conversely, knock-out of Glut1 in T_H_ cells is sufficient to reduce their glycolytic activity, to impair generation of effector T cells, and to reduce inflammatory disease *in vivo* (6, 97).

### Targeting T Cell Metabolism in Inflammatory Skin Disease

Despite considerable recent advances in the field, many insights into the metabolic pathways critical for T cell function derive from *in vitro* T cell assays. However, these are reductionist representations of the complex metabolic situations in the microenvironment *in vivo* (68). Therefore, it remains to be discovered in more detail how exactly T cell immunometabolism is affected in inflammatory skin disease. Nevertheless, by observing how modulating the major metabolic modules affects experimental pathology, some interesting perspectives can be identified and are discussed below.

The mechanisms by which modulation of glycolysis in T cells affects inflammatory pathology has been investigated in some detail. Effector T_H_ cells and CD8^+^ T cells show increased glycolytic activity as compared to naive T cells (16, 67), but not all T_H_ cell lineages have equal glucose metabolism signatures. For instance, T_H_ cells show reduced differentiation into T_H_1 and T_H_17 cells *in vitro* when glycolysis is inhibited, while inducible T_REG_ differentiation is enhanced in low glucose conditions (24, 98). This affects T_EFF_/T_REG_ balance which in turn has been related to reduced pathology in experimental autoimmune encephalomyelitis (98). To what extent modulation of glycolysis can be leveraged to specifically boost or inhibit distinct T_H_ cell subsets is an active area of current research (67). In the context of skin inflammation, it will be crucial to consider the complex interplay between glucose availability in the tissue, glucose consumption by different skin-resident immune, stromal, and epithelial cell populations, and their differential glucose dependence for inflammatory reactions. A summary categorizing key metabolic properties of different T cell subsets in the context of inflammatory skin disease is shown in Table 2.

**Table 2:** T cell subsets in inflammatory skin disease (ISD) and their engagement of key metabolic pathways.

**T cell subset**		**Representative ISD**	**Glycolysis**	**Amino acid metabolism**	**Fatty acid synthesis**	**Mitochondrial biogenesis**
CD4^+^	T_H_1	ACD/Lichen planus	++	+++	+	++
	T_H_2	Atopic Dermatitis	+++	N.D.	N.D.	+
	T_H_17	Psoriasis	++	++	+++	+++
	T_FH_	Lupus/AIBD	+	N.D.	N.D.	++
CD8^+^	CTLs	Severe drug reactions (SJS/TEN)	++	+++	++	+++

*ACD, allergic contact dermatitis; AIBD, autoimmune bullous disease; CTL, cytotoxic T lymphocyte; SJS, Stevens-Johnson-Syndrome; TEN, Toxic epidermal necrolysis. Adapted from Bantug et al. (67) and Eyerich et al. (99)*.

As outlined in more detail above, amino acids are another important substrate for T cell function. Availability of amino acids is likely to change significantly in inflamed skin, which in turn will shape T cell function. T cells require amino acids for protein and nucleic acid synthesis (67) and restriction of amino acids can differentially affect T cell activation via regulation of mTORC1 activation and other pathways (100). In the context of psoriasis, a T_H_17-driven skin disease, activating the amino acid starvation response through halofuginone or restricting amino acid flux into T cells might be of therapeutic interest, since it decreases T_H_17 differentiation, while leaving T_H_1 nor T_H_2 cell polarization unaffected (101, 102). Furthermore, glutamine availability for T cells also impacts differentiation of T_H_ cells. If glutamine is restricted, T_H_ cells polarized under T_H_1 conditions are skewed into a T_REG_ phenotype, whilst again leaving T_H_2 polarization unaffected. Similar findings were made when uptake of glutamine into T cells is inhibited by genetic deletion of the glutamine transporter Slc1a5 (47). Taken together, interference with amino acid metabolism of T cells might be used to improve inflammatory skin disease, particularly psoriasis, as it has been shown to enable therapeutic intervention in multiple T_H_17-driven disease models (47, 67, 101). A number of molecules to inhibit cellular glutamine metabolism are in fact available, some of which are currently being tested clinically in oncological trials (see Table 3) (106, 108).

**Table 3:** Pharmacological modulators of T cell metabolism and their putative therapeutic application in inflammatory skin disease.

**Pathway**	**Drug**	**Effect**	**Target**	**Cellular effects**	**Effects on T cell function/phenotype**	**Putative effect on ISD**	**References**
Glucose metabolism	WZB117	INH	GLUT	Inhibits glucose uptake	Reduced T_H_1/T_H_17 polarization Enhanced iTreg differentiation	Beneficial in ISD with pathologic T_H_17/Treg balance (e.g., PSO)	Macintyre et al. (6), Zhang et al. (93)
	2-DG	INH	Hexokinase (HK)	Inhibits rate limiting step of glycolysis			Shi et al. (98)
	SF2312	INH	Enolase (ENO)	Inhibits rate limiting step of glycolysis			Gemta et al. (103)
Pyruvate metabolism	DCA	INH	Pyruvate dehydrogenase kinase 1 (PDHK1)	Inhibits conversion of pyruvate to acetyl-CoA and glycolysis			Lee et al. (20)
Amino acid metabolism	CB-839	INH	Glutaminase (GLS)	Inhibits glutaminolysis and glutamine-dependent metabolism	Skewing into Treg phenotype	Beneficial in ISD with pathologic T_EFF_/Treg balance (e.g., PSO, AD)	Johnson et al. (104)
	DON	ANT	Glutamine antagonist	Decreased glutaminase activity, IFN-γ production, and proliferation	prevention of transplant rejection (MHC mismatch)	Beneficial in Graft-vs.-host-disease	Lee et al. (20)
	RZ-2994	INH	Serine hydroxymethyl transferase (SHMT)	Inhibits serine biosynthesis !!! Reduced macromolecular biosynthesis and redox balance	Reduced proliferation !!! Impaired (auto-)antigen-specific T cell responses	Benficial in (auto)antigen-driven T cell-mediated ISD (e.g., ACD, AIBD)	Ma et al. (105), Luengo et al. (106)
	HF	INH	Glutamyl-prolyl-tRNA synthetase (EPRS)	Activates amino acid starvation response	Impaired T_H_17 polarization	Beneficial in ISD with pathologic T_H_17 responses (e.g., PSO)	Sundrud et al. (101), Keller et al. (102)
Fatty acid oxidation	Etomoxir	INH	Carnitine palmitoyltransferase 1 (CPT1a)	Inhibits mitochondrial fatty acid β-oxidation	Decreased generation, persistence, and function of T_RM_	Beneficial in TRM-mediated ISDs (e.g., PSO, ACD)	Pan et al. (29, 54)
	GW9662	INH	PPAR-γ	Inhibits upregulation of FABP4/5 and FAO			
Metbolic regulators	Rapamycin	INH	mTORC1 (>mTORC2)	Inhibits translational activity, cell cycle progression, and cell proliferation	Reduced T_H_1/T_H_17 polarization !!! Enhanced iTreg differentiation	Beneficial in ISD with pathologic T_H_17/Treg balance (e.g., PSO)	Pallet et al. (107), Pollizzi and Powell (59)

*ACD, allergic contact dermatitis; AD, atopic dermatitis; AIBD, autoimmune-bullous disease; ANTAG, antagonist; DCA, Dichloroacetate; DON, 6-Diazo-5-oxo-L-norleucine; FABP, fatty acid binding protein; FAO, fatty acid oxidation; GLUT, glucose transporter; HF, halofuginone; INH, inhibitor; ISD, inflammatory skin disease, PSO, psoriasis*.

Modulation of T cell lipid metabolism might also be leveraged to improve inflammatory skin disease. Typically, skin diseases such as psoriasis and atopic dermatitis are characterized by exacerbated activation and proliferation of T cells in the skin (109). Proliferating cells depend on *de novo* fatty acid and cholesterol synthesis as building blocks for the plasma membrane and membranes of organelles. In this regard, there are intriguing differences in the dependence on *de novo* lipogenesis between T_H_17 cells and T_REG_ cells. In particular, if fatty acid synthesis is inhibited by an inhibitor of acetyl-CoA Carboxylase 1 (ACC1, also known as ACACA), T_H_17 cell differentiation and proliferation is reduced while T_REG_ cells are induced (28). ACC1 is an enzyme which catalyzes the carboxylation of acetyl-CoA to malonyl-CoA, the rate-limiting step in fatty acid synthesis. T cells that lack ACC1 show reduced *de novo* synthesis of palmitate and defective T_H_17 cell differentiation. This phenotype can be rescued by exogenous palmitate. Mice harboring ACC1-deficient T cells are less susceptible to EAE which underscores the crucial role of lipid metabolism in pathogenic T_H_17 cell responses and points to a novel therapeutic opportunity in psoriasis (Table 3).

Finally, the particular dependence of skin T_RM_ on β-oxidation of exogenous free fatty acids from the cutaneous environment to fuel their metabolism might represent a weak spot that could be exploited therapeutically (29, 54). In fact, it is increasingly appreciated that chronic and/or aberrant activation of pathogenic T_RM_ represents a key pathogenetic step in the onset and maintenance of many inflammatory skin diseases (3). Psoriasis (110), atopic dermatitis (111), vitiligo (112), cutaneous adverse drug eruption (113), and many other skin diseases have all been shown to be—at least in part—mediated by T_RM_. This provides an elegant explanation for the chronicity of T cell mediated skin diseases and their general tendency to relapse once immunosuppressive treatment is withdrawn. Thus, therapies that not only suppress but actually dislodge pathogenic T_RM_ from their tissue niches in the skin may tackle these diseases at their very root and induce persistent remission. In mouse models, *in vivo* inhibition of mitochondrial β-oxidation with trimetazidine or etomoxir decreased the survival and maintenance of cutaneous T_RM_ and hence show the fundamental feasibility of such therapeutic strategies (29). To what extend such approaches are transferrable to humans will have to be addressed by future studies.

### Crosstalk Between Systemic Metabolism and Skin Inflammation

Both severe psoriasis and severe atopic dermatitis are associated with diseases of the metabolic syndrome (114, 115). Although the reasons for this are incompletely understood, it is known that inflammation can modulate local and systemic metabolism and–vice versa local–that systemic metabolism can profoundly influence immune function (116, 117). Therefore, metabolism and immune function might interact in specific ways to drive aberrant inflammation in inflammatory skin disease. For instance, statins have been proposed to ameliorate psoriasis and their beneficial effects have been attributed to their effects on lipid metabolism in skin, as well as their proclaimed anti-inflammatory and immunomodulatory properties (118). In mice, it has indeed been shown that inhibition of HMG-CoA reductase has an inhibitory effect on T_H_17 cell differentiation (119). Future studies will have to address whether inhibition of HMG-CoA reductase with statins is truly beneficial in inflammatory skin diseases such as psoriasis and whether this potentially beneficial effect is mediated by interference with T_H_17 cell function. In fact, statins may also influence T cell function and inflammation via their inhibitory effect on mitochondrial function. Statins have been shown to inhibit mitochondrial complex III of the respiratory chain (120), leading to reduced generation of mitochondrial reactive oxygen species (mROS). Reduced levels of mROS, in turn, reduce activation of nuclear factor of activated T cells (NFAT) and IL-2 production in T cells (121), thus providing an additional putative mechanism by which statins may alleviate inflammatory skin disease.

In addition, dietary fatty acids from nutrition may also affect systemic immune responses and autoimmunity. In mice, dietary long-chain fatty acids enhance differentiation and proliferation of T_H_1 and T_H_17 cells whereas dietary short-chain FAs expanded gut T_REG_ cells. These nutrition-dependent effects translated into exacerbated EAE via the expansion of pathogenic T_H_1 and/or T_H_17 cell populations (122). Furthermore, a diet high in saturated fatty acids is sufficient to exacerbate psoriatic skin inflammation independent of obesity (123) and this effect seems to be linked to an augmented T_H_17 response in the skin (124). Given that weight loss through diet adaptation can improve pre-existing psoriasis and prevent the onset of psoriasis in obese individuals it can be speculated that the beneficial effects thereof are at least in part mediated through an altered cross-talk between systemic metabolism and local T cell function (125). In addition to fatty acid metabolism, systemic amino acid levels may also affect T cell function in the skin. This has best been studied for the amino acid arginine which is an important modulator of T cell function (48, 126, 127). In animal models, increased dietary arginine enhanced T cell maturation and led to increased contact allergy reactions (128). It is thus possible that systemic and local cutaneous arginine levels may shape the immune response in inflammatory skin diseases.

At any rate, our understanding of the diverse processes that integrate with lymphocyte signaling, gene regulation, and function to shape T cell metabolism is still nascent. A better understanding of the metabolic regulation in T cells and the influence of nutrients in the microenvironment on T cell function will lead to novel insights into T cell function in the context of skin disease. This knowledge may open up new therapeutic approaches in the future.

## Author Contributions

LM has participated in writing the manuscript, researching the literature, and generating the tables and figures. CS has conceived the concept of the review and written the main part of the manuscript. NB has participated in revising the manuscript. This included adding of new text to the manuscript, revising the figures, and editing the existing parts of the manuscript.

### Conflict of Interest

The authors declare that the research was conducted in the absence of any commercial or financial relationships that could be construed as a potential conflict of interest. The reviewer MW and handling editor declared their shared affiliation.
